# Development of a Generic Analysis Method for Isothiazolinones in Consumer Products Prompted by Increased Sensitisation to Benzisothiazolinone

**DOI:** 10.1111/cod.70147

**Published:** 2026-03-26

**Authors:** Anne Farbrot, Jakob Kentson, Hatice Koca Akdeve, Anders Blom, Britt‐Marie Ehn, Naida Babic Mulic, Firoozeh Amirbeagi, Mikael Alsterholm, Lina Hagvall

**Affiliations:** ^1^ Department of Occupational and Environmental Medicine Sahlgrenska University Hospital Gothenburg Sweden; ^2^ Occupational and Environmental Medicine, School of Public Health and Community Medicine, Institute of Medicine University of Gothenburg Gothenburg Sweden; ^3^ Department of Dermatology and Venerology, Region Västra Götaland Sahlgrenska University Hospital Gothenburg Sweden; ^4^ Department of Dermatology and Venereology, Institute of Clinical Sciences, Sahlgrenska Academy University of Gothenburg Gothenburg Sweden; ^5^ Department of Occupational and Environmental Medicine Lund University Lund Sweden

**Keywords:** benzisothiazolinone, contact allergy, HPLC, isothiazolinones, patch testing, preservatives

## Abstract

**Background:**

Isothiazolinones are widely used as preservatives in both cosmetic and non‐cosmetic products. Sensitisation to isothiazolinones other than chloromethylisothiazolinone/methylisothiazolinone (CMIT/MIT), present in the baseline series, is increasing.

**Objectives:**

To investigate the prevalence of sensitisation to benzisothiazolinone (BIT) in comparison to CMIT/MIT and to develop a product‐independent analysis method of isothiazolinones for laboratories with basic instrumentation.

**Patients and Methods:**

Patch testing with BIT and CMIT/MIT was performed in 803 consecutively tested dermatitis patients. A simple and general analysis method for isothiazolinones was developed for HPLC‐UV.

**Results:**

Investigation of 14 products labelled to contain BIT and/or CMIT/MIT (cosmetic and non‐cosmetic) showed concentrations of BIT in non‐cosmetic products of up to 272 ppm. Maximum detected concentrations of CMIT and MIT were 0.3 and 1.5 ppm, respectively. Dichloroctylisothiazolinone and MIT were detected in an unlabelled hobby paint. BIT was a significantly more common cause of sensitisation than CMIT/MIT, at 7.7% and 5.2%, respectively.

**Conclusions:**

Results confirm previous reports indicating that sensitisation to BIT is increasing in dermatitis patients in western Sweden. Analysis of products showed that concentrations of BIT can be several orders of magnitude higher than those of CMIT/MIT in products to which skin contact can be frequent and long lasting.

## Graphical abstract

An analysis method using simple instrumentation was developed to investigate isothiazolinones in cosmetic and household products in patch test investigations. Analysis showed that concentrations of benzisothiazolinone can be several orders of magnitude higher than those of chloromethylisothiazolinone/methylisothiazolinone in products to which skin contact can be frequent and long lasting.

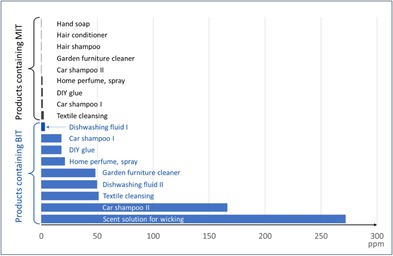

## 1 Introduction

Preservatives are needed in all types of water‐based products to avoid growth of bacteria or fungi. Isothiazolinones are one of the most widely used preservative groups in both cosmetic and non‐cosmetic products, such as chemical/industrial products, household detergents, and water‐based paints [1]. The European baseline series includes methylisothiazolinone (MIT) and chloromethylisothiazolinone (CMIT), as these are present in many cosmetic products. Due to the increased use of and sensitisation to isothiazolinones, CMIT/MIT and MIT were banned in leave‐on products and regulated to 15 ppm in rinse‐off products (Table 1) [2, 3]. The effect of this legislation was clear, as studies reported decreases in sensitisation to CMIT/MIT from peak frequencies of 9%–19% before implementation of the legislation to 1.4%–3.2% after implementation [4, 5]. The regulations are slightly different in other parts of the world (Table 2). In the United States, both BIT and MIT are individually allowed in leave‐on cosmetic products. In Asia, the maximum permissible concentration of MIT is 100 ppm with Japan allowing up to 1000 ppm for the combination of CMIT/MIT in rinse‐off cosmetic products [6, 7].

**TABLE 1:** Maximum permissible concentrations of chloromethylisothiazolinone/methylisothiazolinone (CMIT/MIT), methylisothiazolinone (MIT), benzisothiazolinone (BIT), octylisothiazolinone (OIT), and dichloroctylisothiazolinone (DCOIT) in consumer products available in the EU [2, 3].

Product type	Permissible concentrations (ppm)
Leave‐on cosmetic products	CMIT/MIT banned
	MIT < 15
	BIT banned
	OIT banned
	DCOIT banned
Paint, adhesives, cleaning agents	CMIT/MIT < 15
	MIT < 100
	BIT < 500
	OIT insufficient data
	DCOIT insufficient data
Industrially used biocides	CMIT, MIT and BIT used at > 5000 ppm
	OIT insufficient data
	DCOIT insufficient data

*Note*: For non‐cosmetic products, disclosure of ingredients is not legally required.

**TABLE 2:** Maximum permissible concentrations of chloromethylisothiazolinone/methylisothiazolinone (CMIT/MIT) and methylisothiazolinone (MIT) in cosmetic products in the United States, Canada, Japan, and Korea [6].

Isothiazolinone	Cosmetic products	USA (ppm)	Canada (ppm)	Japan (ppm)	Korea (ppm)
CMIT/MIT	Rinse‐off	15	15	1000	15
	Leave on	7.5	Not allowed	Not allowed	Not allowed
MIT	Rinse‐off	100	100	100	100
	Leave‐on	100	Not allowed	100	Not allowed

However, other isothiazolinones are available for use for manufacturers of water‐based non‐cosmetic products, not regulated in the Cosmetics Directive. Skin contact with these types of products can be extensive. As the use of CMIT/MIT have declined, other isothiazolinones are taking their place. The use of benzisothiazolinone (BIT) has increased in Sweden [8] and BIT was the most widely used isothiazolinone in Danish chemical products in 2014 [9]. BIT has been reported as a moderate sensitiser in animal studies [10]. The maximum level of BIT in indoor and outdoor household products has been regulated to 500 ppm (Table 1). Despite this, sensitisation to BIT is increasing. British and German studies report increases in sensitisation rates to BIT from less than 1% in the 2010s to 3.4%–6.5% in 2020 [4, 11]. BIT sensitisation was found to be significantly more common in metalworkers and painters as well as in male patients compared to female patients [11]. We have previously noted a frequency of sensitisation to BIT of 4.4% in consecutively patch tested dermatitis patients constituting a control group in a study investigating sensitisation to isothiazolinones in painters [12]. Although BIT has now been included in the European baseline series [13], these are, to the best of our knowledge, the only recently published Swedish data from consecutive patch testing with BIT.

Despite the legislation, preservatives can be present in but not declared on both cosmetic and non‐cosmetic products, making it difficult to determine the clinical relevance of positive patch test reactions to isothiazolinones. Apart from BIT, the use of isothiazolinones such as octylisothiazolinone (OIT) and dichloroctylisothiazolinone (DCOIT) in non‐cosmetic products has been found to increase as well [8]. It is therefore important to know more about the presence of commonly used isothiazolinones in products and at which concentrations they are present. Several methods for analysis of isothiazolinones in water‐based products have been published, many also including BIT [14, 15, 16, 17, 18, 19, 20, 21, 22, 23]. These studies have focused on developing a method for a particular type of product, such as cleaning products [17], other types of household products, or cosmetic products [14, 20, 23]. Other methods have been developed for paints and adhesives [21, 24, 25] or for measuring low concentrations of isothiazolinones in air, emitted from paints [26] or disseminated from liquids for air purifiers [24, 27, 28]. The methods are not easily applicable to a range of different types of products in an investigation of a patient's exposure. Nor are the many different analysis methods transferable to other laboratories, due to instrumentation demands and the complexity of sample preparation methods [29]. There is a need for a general method for the analysis of isothiazolinones, applicable to the wide range of products where isothiazolinones are used, and a method that utilises basic instrumentation and straightforward sample preparation.

The aims of the present study were to investigate sensitisation frequencies to BIT in patients consecutively patch tested over two years in western Sweden, and to develop a generally applicable analysis method for quantification of MIT, CMIT, and BIT in a wide range of water‐based cosmetic and non‐cosmetic products. The analysis system was designed to also have capacity for other isothiazolinones, should it be needed.

## 2 Patients and Methods

### 2.1 Analysis Method

#### 2.1.1 Product Selection

The focus was on consumer exposure, with a range of products representing different categories, covering both indoor and outdoor activities, purchased in five different public stores in Gothenburg, Sweden, in September 2021. The condition for inclusion was that the content declaration showed at least one isothiazolinone. A total of 14 products were selected: 2 car shampoos, 1 outdoor textile cleaner, 1 garden furniture cleaner, 1 home perfume air wick solution, 1 air freshener spray, 1 hobby glue, 1 wall paint, 2 dishwashing fluids, 1 hair shampoo, 1 hair conditioner, 1 liquid hand soap, and 1 hair styling gel (Table S1). The isothiazolinones mentioned were MIT, CMIT, and BIT. In addition, an extra sample of hobby paint from a patient investigation of allergic contact dermatitis (ACD) was included. The declaration of content for this product did not mention any isothiazolinones.

#### 2.1.2 Solvents and Substances for Analyses

Methanol (Supelco LiChrosolv hypergrade for LC–MS) and acetic acid (> 99.7%), both used for sample preparation and chromatographic analysis, were purchased from Sigma Aldrich, Germany. Reference substances for quantification were purchased from Sigma Aldrich (St. Louis, USA): CMIT and MIT as a solution in water, a 3:1 mixture (1.1% and 0.4% w/w), while BIT, OIT, and DCOIT all came as 98% purity crystals.

#### 2.1.3 Sample Preparation

Products like soaps and detergents that lack polymeric constituents were diluted before analysis (procedure A), while glue and paint products were dissolved, diluted, and filter centrifuged (procedure B). Both procedures used a solution of methanol/deionised water, 45/55 (vol.) + 0.5% (vol.) acetic acid for isothiazolinone extraction and sample dissolution.

Procedure A: 5 g of product was weighed into a 100 mL beaker. The weight was recorded to the second decimal and 50 mL of the sample extraction/dilution solution was added to the beaker. The samples were sonicated for 10 min to mediate extraction and dissolution of the products, whereafter the samples were completely transferred to a 100 mL volumetric flask and volume adjusted with sample extraction/dilution solution. The final dilution was approximately 1/20, depending on the initial weight. A portion of 30–40 mL was transferred to 50 mL Falcon tubes (Corning Science, Mexico) and centrifuged at 4500 rpm for 10 min at room temperature (rt). Finally, 1.5 mL of each product solution was transferred into HPLC screw‐cap vials.

Procedure B (glue and paint): 2.5 g of product was weighed into a 50 mL Falcon tube, exact weight noted, and 5.0 mL of sample extraction/dilution solution was added. The product was manually dissolved by stirring before another 5.0 mL of sample extraction/dilution solution was added. The solution was centrifuged for 30 min at 4500 rpm (rt). The supernatant was transferred to a 25 mL filter centrifuge tube (0.45 μm PVDF‐filter, Thermo Scientific, USA) and centrifuged for 30 min at 4500 rpm (rt), whereafter a portion of 1.5 mL was transferred into HPLC vials.

Products were stored shortly at room temperature before samples were taken for analysis from freshly opened bottles and containers. Sample extracts were refrigerated and shielded from light before analysis. Isothiazolinone stability in opened containers was not included in the scope of the present study.

#### 2.1.4 Analytical Instrument System

Analysis was performed using an Agilent 1260 HPLC system with a binary pump, column oven, and scanning UV‐detector (diode array, DAD). Gradient elution with mobile phases A: Water 95%, MeOH 5%, B: MeOH 100%. Both with an addition of acetic acid, 4 mL/L. The gradient used is presented in Table 3.

**TABLE 3:** HPLC gradient programme.

Time	A%	B%	Flow (mL/min)
0:00	99	1	1.3
13:00	5	95	1.3
16:00	5	95	2.0
17:00	99	1	1.5
20:00	99	1	1.3

The HPLC‐column was a 4.6 × 100 mm 2.5 μm EternityXT‐PhenylHexyl phase (Kromasil, Nouryon, Sweden) with protective C_8_ pre‐column (Zorbax, Agilent, USA) with the temperature kept at 40°C. Operating pressure was in the range 190–300 bar. Injection volume was 10 μL and detection was performed at optimised wavelengths: 275 nm for MIT, CMIT, OIT and DCOIT and 320 nm for BIT using 10 nm bandwidth and no background correction.

Stock reference solutions of BIT, OIT and DCOIT were prepared in methanol. The CMIT/MIT solution was used as it was. Calibration standards of 10, 50 and 100 μg/mL were obtained through dilution with the dilution/extraction solution of methanol/deionised water, 45/55 (vol.) + 0.5% (vol.) acetic acid.

#### 2.1.5 Method Validation

The extraction efficiency of isothiazolinones from products was verified by repeatedly (4 times) analysing two different product types: a car shampoo and a wall paint. The limit of detection (LOD) was determined as three times the background signal, measured in a blank sample. The blank was obtained by performing extraction procedure A without the addition of product. The limit of quantification was set to 10 times the LOD. The calibration covered the range of concentrations in the different products and was within the linear range of the detector.

### 2.2 Patch Testing

#### 2.2.1 Patch Test Preparations

Patch test preparations of CMIT/MIT (0.215% aq., 0.015% CMIT, 0.2% MIT, CAS 55965–84‐9) and BIT (0.1% pet. CAS 2634‐33‐5) were purchased from Chemotechnique Diagnostics, Vellinge, Sweden.

#### 2.2.2 Test Population

The test population included all patients referred to the Department of Dermatology and Venereology at Sahlgrenska University Hospital, Gothenburg, Sweden, for suspected allergic contact dermatitis and subsequently patch tested during the period 1 January 2022 to 31 December 2023 (Table 4). All patients were tested with CMIT/MIT as part of the Swedish baseline series with the addition of BIT.

**TABLE 4:** Age and sex distribution in the test population consisting of all consecutively patch tested patients at the Department of Dermatology and Venereology, Sahlgrenska University Hospital, in the years 2022–2023.

Year	Patients (*n*)	Age (mean ± SD)	Male (*n* (*%*))	Age males(mean ± SD)	Female (*n* (*%*))	Age females (mean ± SD)
2022	387	45 ± 19.0	121 (31)	47 ± 19.7	266 (69)	45 ± 18.7
2023	416	44 ± 17.5	117 (28)	43 ± 16.5	299 (72)	44 ± 17.9
Total	803		238		565	

#### 2.2.3 Patch Testing Procedure

The preparations were applied in 8 × 8 mm IQ Ultra chambers mounted on a hypoallergenic surgical tape (Chemotechnique Diagnostics, Vellinge, Sweden). The applied dose of hapten was 20 μL for CMIT/MIT and 25 mg for BIT. The chambers were applied on the back of patients under occlusion for 48 h. Patients removed the patches themselves at 48 h. Patch test readings were performed on day (D)3 and D7, by a dermatologist and a lab technician trained in patch test reading. Patients were routinely asked to report late reactions beyond the second reading. If a late reaction occurred, the patient would be offered an appointment for an extra patch test reading. Readings were classified according to the European Society of Contact Dermatitis (ESCD) guidelines, and positive tests were evaluated for clinical relevance by a dermatologist [30].

### 2.3 Statistical Analysis

Total frequencies of contact sensitisation to CMIT/MIT and BIT were compared using the exact binomial test. Frequencies of sensitisation to BIT in men and women were compared using Fisher's exact test.

### 2.4 Ethics

The study was approved by the Regional Ethical Committee of Gothenburg and performed in accordance with the ethical standards in the Declaration of Helsinki. Participants provided informed written consent before participation.

## 3 Results

### 3.1 Analysis Method

**FIGURE 1:**
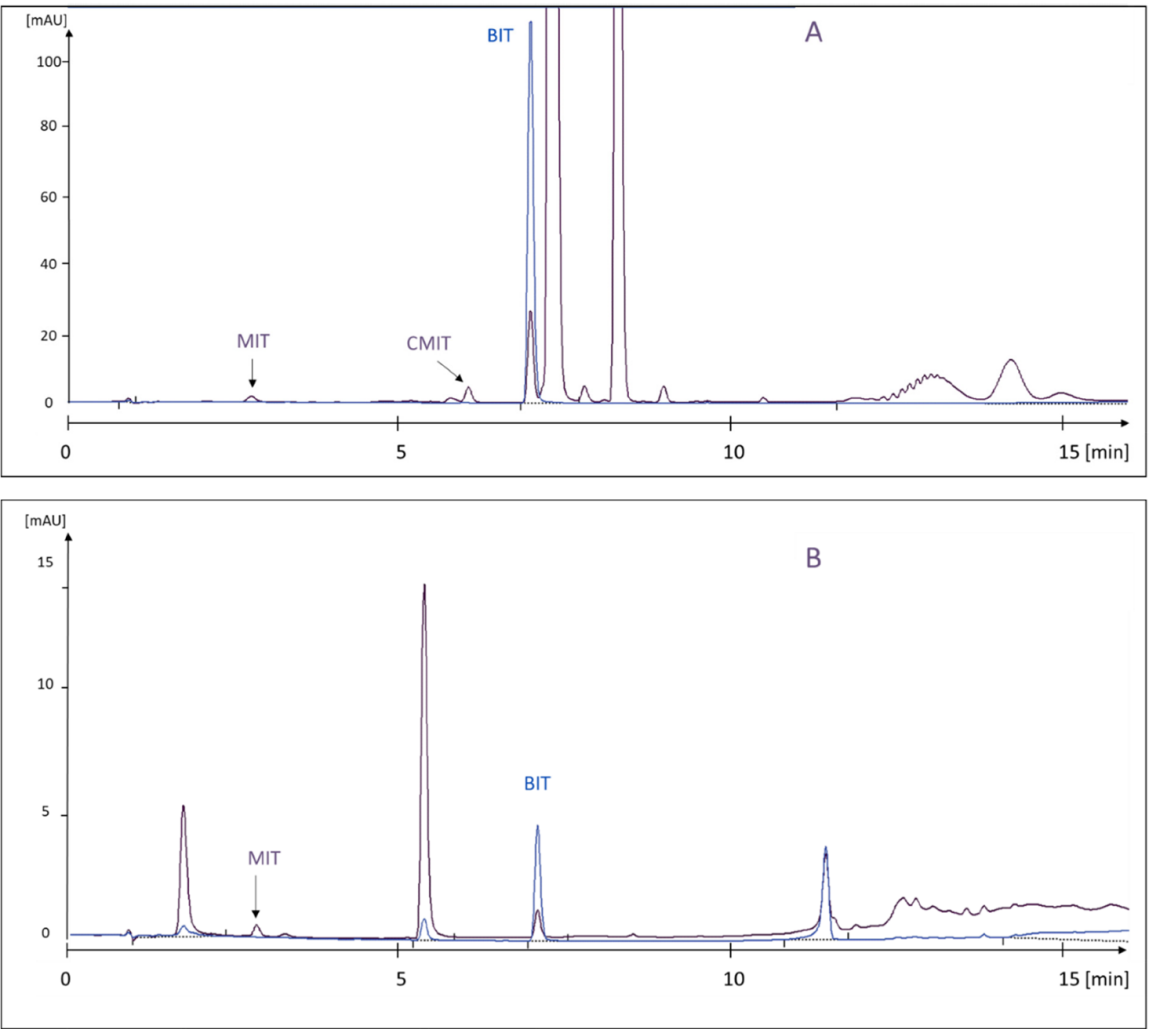
Chromatograms of (A) indoor paint, (B) garden furniture cleaner, both with isothiazolinones well separated from matrix constituents. BIT, benzisothiazolinone; CMIT, chloromethyl isothiazolinone; MIT, methylisothiazolinone.

The precision of the extraction procedures showed relative standard deviations of < 10% for both extraction procedures (Section 2.3) based on repeated extractions of two different types of products. The effectiveness of the extractions was not further investigated. The chromatographic analysis on the phenyl‐substituted column could clearly separate isothiazolinones from matrix interferences, thereby facilitating unambiguous quantification (Figure 1A,B). Furthermore, the limits of quantification (LOQ) in products were well below the permissible levels of MIT, CMIT (15 ppm), and BIT (500 ppm) using both extraction procedures. LOQ was in the range 0.005–0.1 ppm (μg/g) for MIT through to DCOIT.

**FIGURE 2:**
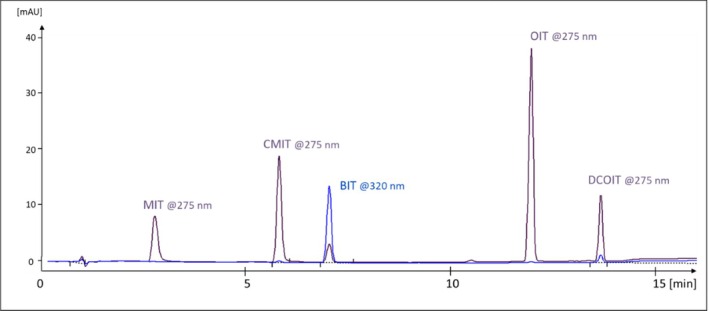
Chromatogram of standards methylisothiazolinone (MIT) 25 μg/mL, chloromethyl isothiazolinone (CMIT) 75 μg/mL, benzisothiazolinone (BIT) 50 μg/mL, octylisothiazolinone (OIT) 50 μg/mL and dichloroctylisothiazolinone (DCOIT) 50 μg/mL, all in methanol. Detection wavelengths indicated.

The chromatographic method was originally set up for MIT, CMIT, and BIT with a gradient that continues with increasing solvent strength, also after BIT elutes at 7.0 min. This was necessary for analyses of products containing hydrophobic (less water‐soluble) components found in glues and paints, see example in Figure 1A. Thereby the method also works for the more hydrophobic isothiazolinones octylisothiazolinone (OIT) and dichloroctylisothiazolinone (DCOIT) (Figure 2).

Retention times for the other IT were MIT: 2.8 min, CMIT: 5.9 min, OIT: 12.0 min, DCOIT: 13.6 min.

### 3.2 Isothiazolinone Concentrations in Cosmetic and Household Products

**FIGURE 3:**
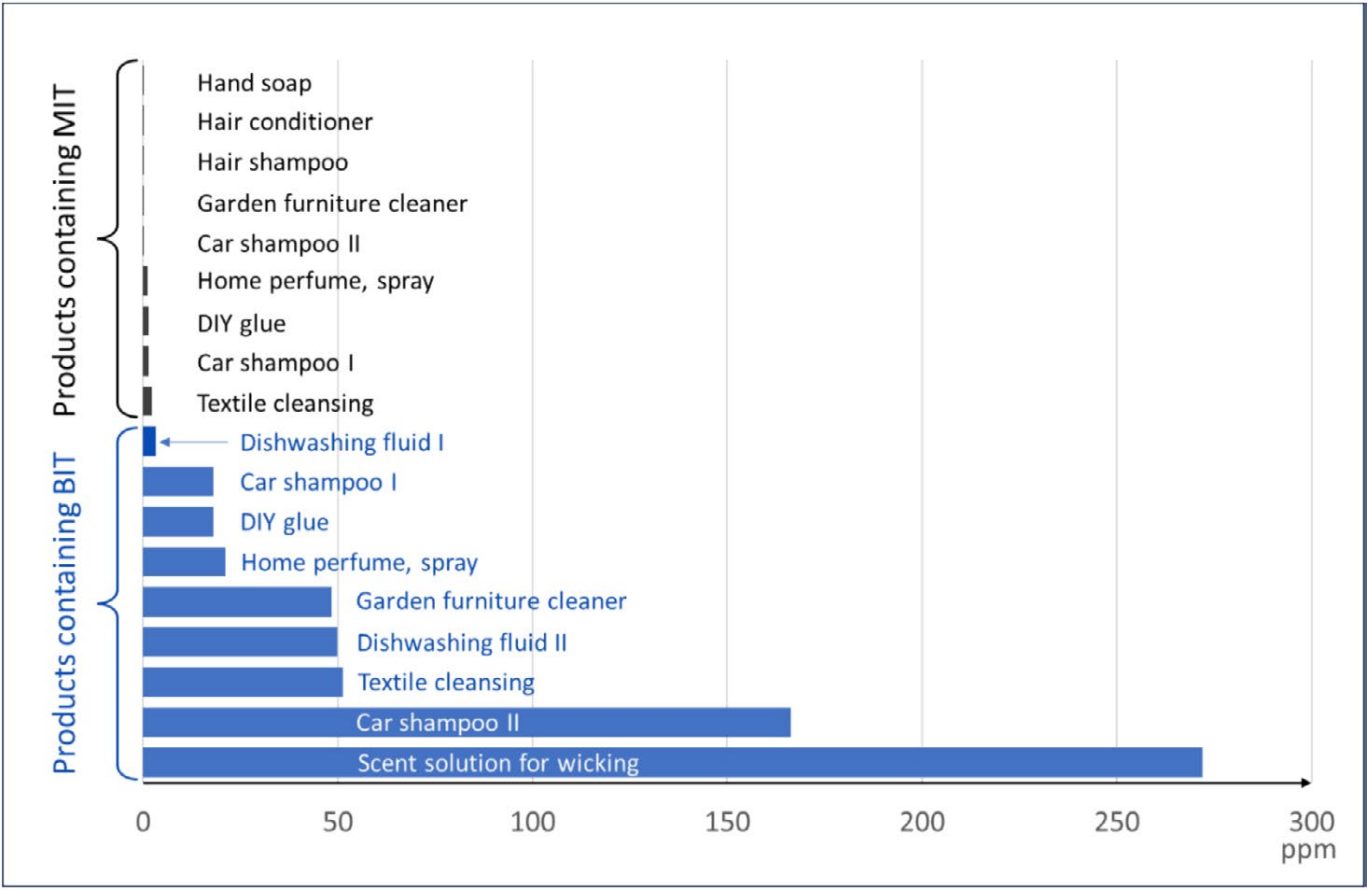
Benzisothiazolinone (BIT) and methylisothiazolinone (MIT) content in investigated cosmetic and household products. A complete list of concentrations is found in Table S1.

Analyses display very different amounts of isothiazolinones among the products, visualised in Figure 3. Of the 14 analysed products, 9 contained MIT, 9 contained BIT, and 6 products contained both MIT and BIT. BIT was not detected in any cosmetic products. There were just 6 products declared to contain CMIT, with only 3 showing measurable amounts (0.3 ppm). All results fall within the respective regulations with MIT and CMIT in the range 0.02–2.3 ppm (limit 15 ppm) and BIT concentrations ranging from 3 to 270 ppm (500 ppm limit).

**FIGURE 4:**
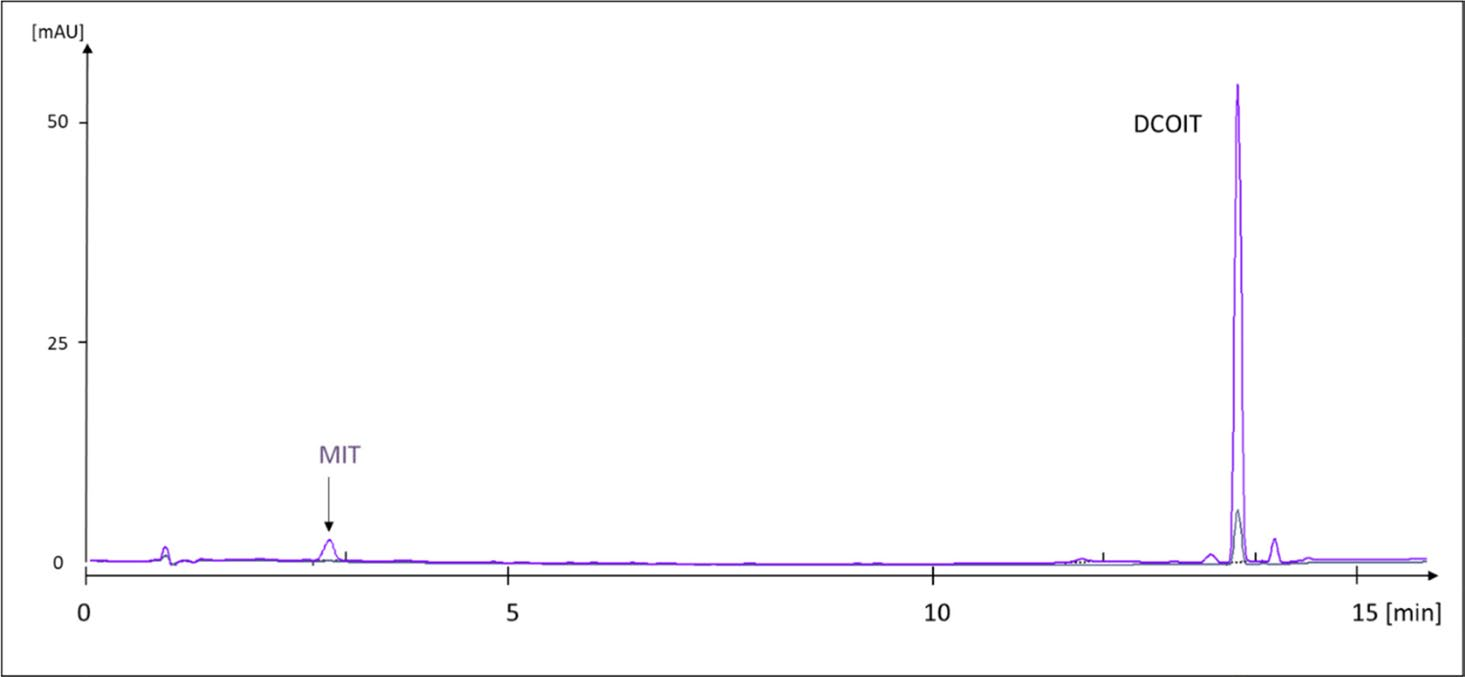
Chromatogram of common hobby paint with no isothiazolinone labelling. DCOIT, dichloroctylisothiazolinone; MIT, methylisothiazolinone.

In addition to the targeted collection of products declared to contain MIT, CMIT, and BIT, a patient's hobby paint with no isothiazolinone labelling was analysed because of the patient's patch test reaction to BIT (+). The analysis revealed 5 ppm of MIT and 340 ppm of DCOIT (Figure 4).

### 3.3 Sensitisation to CMIT/MIT and BIT

**FIGURE 5:**
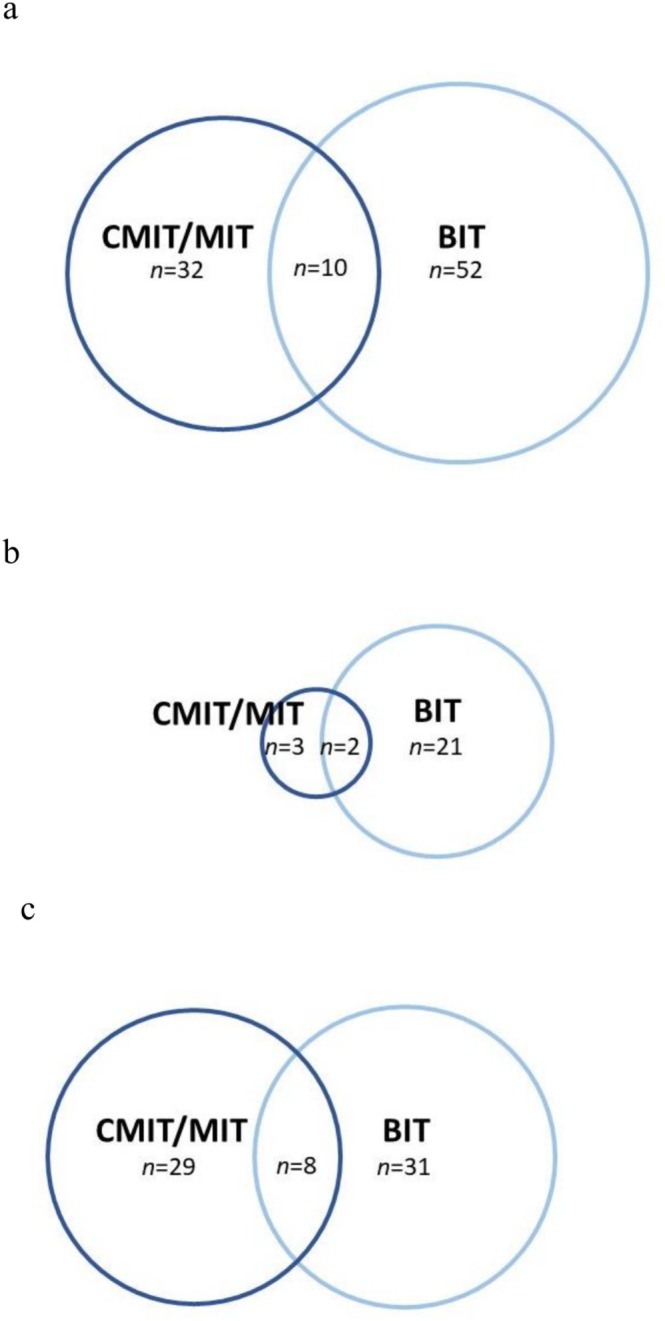
Venn diagrams of all positive patch tests to chloromethylisothiazolinone/methylisothiazolinone (CMIT/MIT, 0.215% aq) (*n* = 42) and benzisothiazolinone (BIT, 0.1% pet.) (*n* = 62) in all patients tested at the Department of Dermatology and Venereology, Sahlgrenska University Hospital, in the years 2022–2023 (A), and the distribution of positive tests in men (B) and women (C). The total number of patch tested patients was *n* = 803 (men *n* = 238, women *n* = 565).

A total of 803 patients (565 women, 238 men) were patch tested. Positive patch test reactions to BIT were more prevalent than positive reactions to CMIT/MIT, 7.7% (62/803) and 5.2% (42/803), respectively (*p* = 0.038) (Table 5). The distribution of strength of positive reactions as well as doubtful and irritant reactions to CMIT/MIT and BIT, respectively, is presented in Table 6. Among patients reacting to CMIT/MIT, 69% showed a strong reaction (++/+++) whereas 13% of patients reacting to BIT showed a strong reaction. Concomitant reactivity to CMIT/MIT and BIT were found in only 10 patients, corresponding to 1.2% of the total test population or 10.6% of patients reacting to at least one of CMIT/MIT and BIT (Figure 5A). The ratio of BIT to CMIT/MIT sensitisation was higher in men (9.7%, 23/238 vs. 2.1%, 5/238) than in women (6.9%, 39/565 vs. 6.5%, 37/565) but sensitisation to BIT was not significantly more common in men (*p* = 0.19, Fisher's exact test) (Figure 5B,C).

**TABLE 5:** Positive patch test reactions to chloromethylisothiazolinone/methylisothiazolinone (CMIT/MIT) (0.215% aq.) and/or benzisothiazolinone (BIT) (0.1% pet.) in 803 consecutively tested patients at the Department of Dermatology and Venereology, Sahlgrenska University Hospital, in the years 2022–2023.

	CMIT/MIT		BIT		CMIT/MIT + BIT	
	*n*	% (95% CI)	*n*	% (95% CI)	*n*	% (95% CI)
2022						
Male	2	1.7 (0.2–5.8)	12	9.9 (5.2–17)	0	0 (0.0–3.0)
Female	21	7.9 (5.0–12)	24	9.0 (5.9–13)	7	2.6 (1.1–5.3)
All	23	5.9 (3.8–8.8)	36	9.3 (6.6–13)	7	1.8 (0.7–3.7)
2023						
Male	3	2.6 (0.5–7.3)	11	9.4 (4.8–16)	2	1.7 (0.2–6.0)
Female	16	5,4 (3.1–8.5)	15	5.0 (2.8–8.1)	1	0.3 (0.0–1.8)
All	19	4.6 (2.8–7.0)	26	6.3 (4.1–9.0)	3	0.7 (0.1–2.1)
2022 + 2023						
Male	5	2.1 (0.7–4.8)	23	9.7 (6.2–14)	2	0.8 (0.1–3.0)
Female	37	6.5 (4.7–8.9)	39	6.9 (5.0–9.3)	8	1.4 (0.6–2.8)
All	42	5.2 (3.8–7.0)	62	7.7* (6.0–9.8)	10	1.2 (0.6–2.3)

*Note*: * *p* = 0.038, exact binomial test.

**TABLE 6:** Distribution of strength of positive reactions to CMIT/MIT (0.215% aq.) and BIT (0.1% pet.) as well as doubtful (?) and irritant (IR) reactions in all patients with positive reactions to CMIT/MIT and BIT, respectively.

	CMIT/MIT					BIT				
	*n* pos	*n* +	*n* ++/+++	*n* ?	*n* IR	*n* pos	*n* +	*n* ++/+++	*n* ?	*n* IR
Male	5	2	2/1	0	0	23	21	2/0	0	0
Female	37	11	13/13	0	0	39	33	5/1	0	0
All	42	13	15/14	0	0	62	54	7/1	0	0

## 4 Discussion

### 4.1 Analysis Method

Many laboratories are using high‐end techniques (U)HPLC‐MS(‐MS) [29] which will undoubtedly work well for the products studied. However, the use of conventional HPLC equipment with UV detection presented here is efficient in analysing isothiazolinone content in a wide range of common everyday products, containing as little as 0.05% of the allowed amounts.

It is an advantage to be able to employ one overall procedure for efficient handling of many different types of products. The composition of the waterbased products under investigation, the generous amounts of product available and the high concentrations of the isothiazolinones make them amenable to basic extraction, dilution, and filtering procedures. Therefore, it is possible to utilise basic laboratory equipment. For products with a high viscosity, there is a need to use filtering, which is conveniently done in a centrifuge, adding an extra step to the sample preparation procedure.

We were able to use sample preparation solvent containing 45% methanol for all the investigated product types. The use of organic solvent was not needed for MIT and CMIT, but necessary in order to extract and dissolve BIT, OIT, and DCOIT. A key measure here was to employ a phenyl substituted column, which provides superior retention for MIT (Figure 2) and thereby unadulterated peak shape. In contrast, methods using C_18_‐ or similarly substituted stationary phases will have MIT eluting at the very beginning with a broadened peak due to the high portion of methanol in the sample, whereby quantification becomes both difficult and less sensitive. At the other end of hydrophobicity, DCOIT would be strongly retained on a C_18_‐column, leading to longer analysis time and excessive use of solvents. Considering the increased use of other isothiazolinones such as OIT and DCOIT, it is important to be able to simultaneously screen for many isothiazolinones effectively and efficiently, which the present method has shown.

### 4.2 Isothiazolinone Concentrations in Cosmetic and Household Products

All concentrations of MIT were under the regulated limit for cosmetic products of 15 ppm (Figure 3). The measured concentrations of BIT revealed an extensive variation but did not exceed the limit for BIT in consumer products (500 ppm). In a study from 2015, MCIT, MIT, and BIT content was analysed in cosmetic and consumer products on the Belgian market [31]. There, higher concentrations of MIT were found in cosmetic products compared to the present study, and BIT was found only in floor cleaning products. Although both studies are small, data indicate the use of BIT instead of MIT in common consumer products such as dishwashing liquid.

As the regulations differ between different types of products, that is, cosmetic products versus consumer products, exposure to isothiazolinones can be high, especially if exposure occurs from several types of products simultaneously. Further, as labelling of BIT is not mandatory on these types of products, investigation of relevance of BIT contact allergy as well as avoidance of BIT containing products is difficult for BIT allergic patients.

### 4.3 Sensitisation to BIT and CMIT/MIT

The sensitisation rate to BIT in the present study (Table 5) was higher than our previous results, where 4.4% of patients showed positive reactions to BIT [12]. The increasing sensitisation rate of BIT in western Sweden is in line with recently published results from Europe [4, 11]. However, an increase was also observed in the sensitisation rate to CMIT/MIT compared to our previous study, where 1.3% of patients showed positive reactions [12]. Albeit a smaller study population in our previous study (385 patients), the results of the present study point to an increase in positive reactions to CMIT/MIT. This is most probably an effect of the increase in MIT concentration in the test preparation of CMIT/MIT in the Swedish baseline series, updated in 2021. However, differences in frequencies of contact allergy to BIT between genders are difficult to analyse as the group of patch tested men is small. The optimal mixture and test concentrations for patch testing CMIT/MIT have been thoroughly studied [32, 33], however, this has not been performed for BIT. As only a small proportion of BIT cases showed strong reactions, it is possible that the current test concentration used is too low. Assessments of clinical relevance of BIT reactions in patch testing are difficult since information about BIT content in products is hard to find or erroneous. Thorough studies of both optimal patch test concentrations of BIT and clinical relevance of BIT reactions should be performed, the latter now possible using the analytical method presented in this paper.

The majority of cases of sensitisation to BIT or CMIT/MIT did not show concomitant reactivity to the other isothiazolinones (Figure 5). Patch testing with CMIT/MIT 0.215% detected only 16% of cases of contact allergy to BIT. There are indications of cross reactivity between MIT and BIT in mice [34], however, opposite results have been found in clinical studies of concomitant positive reactions to BIT and MIT [11]. The results of the present study support the results of these clinical studies. Although all isothiazolinones share a common isothiazolinone ring structure, investigated isothiazolinone derivatives differ markedly in structure of the side chains, causing these isothiazolinone derivatives to differ greatly in chemical properties and three‐dimensional structure perceivable to immunocompetent cells. This is also shown in the differences in solubility and polarity, posing challenges in the analytical method. Concomitant reactions are instead believed to be the consequence of concomitant exposure. As shown in Figure 3, BIT and MIT were in many cases detected in the same product. Although BIT is a weaker sensitiser than MIT, with an EC3 value of 1.9% in the local lymph node assay in mice, compared to 0.4% for MIT [34], the difference does not seem to allow for the high concentrations of BIT permitted in household products, as sensitisation rates to BIT are increasing in Europe. Further, products can be incorrectly labelled, as was the case with the hobby paint analysed as an example product from a patient investigation (Figure 4). It would have been impossible to give constructive advice to the patient regarding secondary prevention without results from chemical analysis of the hobby paint.

### 4.4 Conclusion

Patch test results confirm previous reports indicating that sensitisation to BIT is increasing in dermatitis patients in western Sweden. A robust analysis method using simple extraction and detection methods has been developed, which can be used to investigate isothiazolinone content in a wide variety of products. Using the method, analysis of products showed that concentrations of BIT can be several orders of magnitude higher than those of CMIT/MIT in products to which skin contact can be frequent and long lasting. Taken together, the results indicate that present regulation of BIT in consumer products does not protect consumers against either sensitisation or allergic contact dermatitis to BIT.

## Author Contributions

**Anne Farbrot:** conceptualization, methodology, writing – original draft, writing – review and editing. **Jakob Kentson:** investigation, writing – original draft, writing – review and editing, methodology. **Hatice Koca Akdeve:** investigation, writing – review and editing. **Anders Blom:** investigation, writing – review and editing. **Britt‐Marie Ehn:** investigation, writing – review and editing. **Naida Babic Mulic:** investigation, writing – review and editing. **Firoozeh Amirbeagi:** investigation, collocation of data, writing – review and editing. **Mikael Alsterholm:** investigation, writing – review and editing. **Lina Hagvall:** conceptualization, methodology, visualization, writing – review and editing, writing – original draft, project administration.

## Conflicts of Interest

The authors declare no conflicts of interest.

## Supporting information


**Table S1:** Concentrations of methylisothiazolinone (MIT), methylcholoroisothiazolinone (CMIT) and benzisothiazolinone (BIT) in the analysed products given in mmol/l (mM) and as ppm.

## Data Availability

The data that support the findings of this study are available on request from the corresponding author. The data are not publicly available due to privacy or ethical restrictions.
