# Supplementary material for: Development of a Generic Analysis Method for Isothiazolinones in Consumer Products Prompted by Increased Sensitisation to Benzisothiazolinone
Source: Contact Dermatitis. 2026 Mar 26;95(1):33–42. doi: 10.1111/cod.70147 (PMC13238301; doi:10.1111/cod.70147)
Supplement: Supplementary file 1 — Table S1: Concentrations of methylisothiazolinone (MIT), methylcholoroisothiazolinone (CMIT) and benzisothiazolinone (BIT) in the analysed products given in mmol/l (mM) and as ppm. [file COD-95-33-s001.docx]

# Supplementary Information

**Table S1.** Concentrations of methylisothiazolinone (MIT), methylcholoroisothiazolinone (CMIT) and benzisothiazolinone (BIT) in the analysed products given in mmol/litre (mM) and as ppm

| PRODUCT | EXTRACTION PROCEDURE | MIT  (mM) | MIT  (ppm) | CMIT  (mM) | CMIT  (ppm) | BIT  (mM) | BIT  (ppm) |
| --- | --- | --- | --- | --- | --- | --- | --- |
| CAR SHAMPOO I | A | 0.013 | 1.5 | ND | - | 0.12 | 18 |
| CAR SHAMPOO II | A | 0.002 | 0.23 | ND | - | 1.1 | 166 |
| OUTDOOR TEXTILE CLEANer | A | 0.02 | 2.3 | NA | - | 0.34 | 51 |
| GARDEN FURNITURE CLEANER | A | 0.002 | 0.23 | NA | - | 0.32 | 48 |
| INDOOR PAINT | B | 0.086 | 10 | 0.013 | 2 | 1.5 | 227 |
| GLUE | B | 0.012 | 1.4 | 0.002 | 0.3 | 0.12 | 18 |
| DISHWASHING FLUID I | A | NA | - | NA | - | 0.021 | 3.2 |
| DISHWASHING FLUID II | A | NA | - | NA | - | 0.33 | 50 |
| SHAMPOO | A | 0.0007 | 0.08 | 0.002 | 0.3 | NA | - |
| CONDITIONER | A | 0.0005 | 0.06 | 0.002 | 0.3 | NA | - |
| LIQUID HAND SOAP | A | 0.0002 | 0.02 | ND | - | NA | - |
| HAIR STYLING GEL | B | ND* | - | NA | - | NA | - |
| HOME PARFUME SPRAY | A | 0.011 | 1.3 | NA | - | 0.14 | 21 |
| AIR WICK SCENT SOLUTION | A | NA | - | NA | - | 1.8 | 272 |

ND: Not detected. Below LOD but mentioned on the declaration of content
NA: Not applicable, the product did not contain the IT according to the declaration of content
*Not detected due to interferences from the matrix
